# Roots of *Apium graveolens* and *Petroselinum crispum*—Insight into Phenolic Status against Toxicity Level of Trace Elements

**DOI:** 10.3390/plants10091785

**Published:** 2021-08-27

**Authors:** Danijela Arsenov, Milan Župunski, Slobodanka Pajević, Ivana Nemeš, Nataša Simin, Abdullah M. Alnuqaydan, Malcolm Watson, Abdulaziz A. Aloliqi, Neda Mimica-Dukić

**Affiliations:** 1 Department of Biology and Ecology, Faculty of Sciences, University of Novi Sad, 21000 Novi Sad, Serbia; milan.zupunski@dbe.uns.ac.rs (M.Ž.); slobodanka.pajevic@dbe.uns.ac.rs (S.P.); 2 Department of Chemistry, Faculty of Sciences, Biochemistry and Environmental Protection, University of Novi Sad, 21000 Novi Sad, Serbia; ivana.nemes@dh.uns.ac.rs (I.N.); natasa.simin@dh.uns.ac.rs (N.S.); malcolm.watson@dh.uns.ac.rs (M.W.); neda.mimica-dukc@dh.uns.ac.rs (N.M.-D.); 3 Department of Medical Biotechnology, College of Applied Medical Sciences, Qassim University, Buraydah 51452, Saudi Arabia; aaalieky@qu.edu.sa

**Keywords:** vegetables quality and pollution, antioxidative response, polyphenols, metal/metalloid accumulation, health risk assessment

## Abstract

Celery (*Appium graveolens* L.) and parsley (*Petroselinum crispum* (Mill.) Fuss) are herbs utilized in the everyday diet as spices and culinary flavorings, often used in the chemical and medicinal industries. Despite the knowing benefits of different plants from the Apiaceae family, their chemical composition is closely associated with various extrinsic factors. Environmental loading with trace elements (TEs) can modify a plant’s metabolic pathways, change bioactive compounds production, cause plant pollution, and consequently provoke human health issues. Therefore, we established this research aiming to unravel the linkage between TEs accumulation and phenolic status in celery and parsley. Higher As, Cd, and Ni levels were observed in celery, which was followed by greater DPPH^∙^ radical scavenging activity and higher coumarins content. Contrary, parsley accumulated chromium to a greater extent, was richer in flavonoids, apigenin, and its glucosides. No significant difference between species was found in total phenolic contents, where ferulic and chlorogenic acid dominated in both species. A direct relationship between TEs and selected secondary metabolites was proven by the standardized major axis model. Besides abundant bioactive compounds, analyzed plant species showed a moderate hazard index in the children population, since the hazard index was higher than 1. Therefore, future perspectives should be turned towards the production of genotypes with a lower potential for toxic elements accumulation, so the health benefits of plant food will be more prominent.

## 1. Introduction

In an era of rapid development of a diverse range of acute and chronic diseases, increased health awareness associated with daily immunity-boosting with fresh and healthy superfoods is imperative. It is well known that lifestyle and diet are among the major factors influencing human health. Therefore, enhanced vegetable consumption is strongly recommended by the World Cancer Research Fund (WCRF) and the American Institute for Cancer Research (AICR), as such a diet can mitigate the risk of the onset of various diseases. [1]. Since ancient times, species from the Apiaceae family have been recognized as aromatic plants, possessing a number of beneficial effects on human wellbeing, thus they have been historically used in traditional medicine. Among the Apiaceae family, celery and parsley are herbs cultivated worldwide, utilized in the everyday diet as spices and culinary flavorings, and are often used in the chemical and medicinal industries [2,3,4]. Both species are known to possess a plethora of nutrients and phytochemicals; they are rich in vitamins, minerals, β-carotene, proteins, phenols, flavonoids, essential oils, and antioxidants [5,6,7], which makes them favorable as dietary components and nutraceuticals. Furthermore, celery and parsley can be labeled as functional foods, since they can deliver a wide range of additional benefits beyond the basic nutrition requirements, and can serve in the prevention or delay of certain diseases [8]. In traditional medicine, celery is used as a diuretic and antirheumatic; in addition, it is clarified as a species rich in volatile terpenoids and phenolics, mostly coumarins and flavonoids [2,9,10]. It is also known that celery could possess healing effects against hypertension, diabetes, and cardiovascular disorders, based on its antioxidant, antibacterial, and antifungal properties. Similar properties are ascribed to parsley, as it is stated to possess carminative, antispasmodic, diuretic, antirheumatic, antipyretic, and antimicrobial activity [5]. In addition, parsley can be utilized for menstrual disorders, gastrointestinal problems, and for breath cleaning [4,11]. All of these properties make plants from the Apiaceae family highly appreciated in the human diet and attractive for exploration and investigation by the food and pharmaceutical industry. Despite the known benefits of celery and parsley, their metabolic profile and chemical composition are closely associated with various extrinsic stimuli. Different environmental factors such as drought, extreme temperature, and heavy metals pollution can cause shifts in the metabolic activities of plants, thus representing the main driving forces, as well as constraints for plant primary and secondary metabolism [12]. Under perturbated environmental conditions, plants can modify their key metabolic pathways by elevating the production of bioactive compounds, which can serve as protectors of imposed stresses. At the same time, energy investment in secondary metabolism can provoke shifting in the allocation of resources, at first place carbohydrates, which can consequently lead to plant growth retardation. In addition, environmental loadings with trace elements (TEs) have become severely increased in recent years and were mostly provoked by intensive anthropogenic activities [12]. This is especially pronounced when it comes to toxic elements, which have gained unprecedented importance on a global scale, considering the fact that TEs can be accumulated in plants, used in the human diet, and can endanger food security and human health. Based on their physiological properties, well-developed roots, lush aerial biomass, annual growth, celery, and parsley represent potential candidates for high metals accumulation [13,14]. Roba et al. [15] defined parsley as a species with high accumulation potential and indicated that its regular use on a daily basis can lead to potential health issues for local residents. Additionally, our previous study confirmed that celery has an even higher potential to accumulate various toxic/potentially toxic elements (TEs) than parsley, especially in Cd-polluted soil [16]. Overall, both species can serve as sources for metal removal, while at the same time possess plenty of nutritive values beneficial for human health.

In light of that contrast, we established this research aiming to unravel the linkage between TEs accumulation and plant secondary metabolism. With that purpose, we analyzed the concentrations of the most abundant TEs (Cr, As, Ni and Cd), phenolic profiles, and antioxidant potential of commercially cultivated celery and parsley aiming to point out TEs and to what extent they can modify plant metabolic responses. Therefore, we used the standardized major axis (SMA) regression model, which is a useful tool in the analysis of two factors, assuming variation in both variables [17]. To the best of our knowledge, this is the first study that will utilize this kind of approach, therefore, the results can possibly extend the current knowledge of the relationship between TEs present in celery and parsley with their metabolic profile.

## 2. Results and Discussion

### 2.1. Trace Elements Accumulation

**Figure 1:**
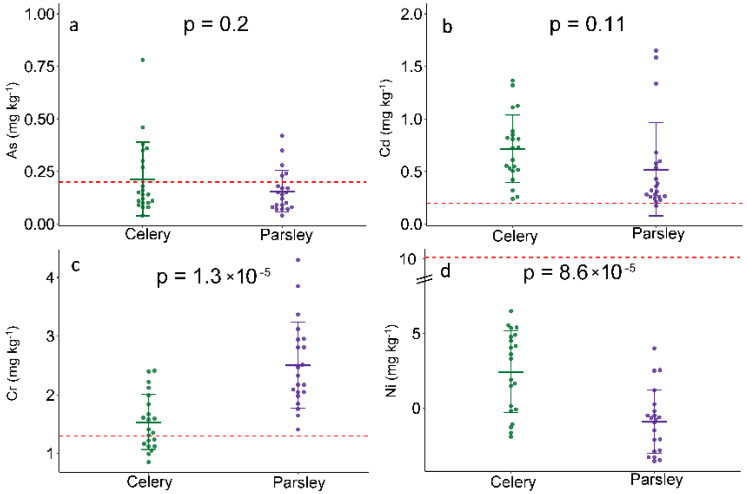
Trace elements concentration in celery and parsley root: (**a**) arsenic; (**b**) cadmium; (**c**) chromium; (**d**) and nickel content (mg/kg dry mass). Data are presented as the mean ± standard deviation. Unpaired *t*-test was used for comparing the means between species (*n* = 21) and the *p*-value is assigned to each plot, *p* ≤ 0.05 has been considered as significant. Red dashed line indicates maximum allowable concentration (MAC) for the corresponding element: As–0.2; Cd–0.2; Cr–1.3; Ni–10 mg/kg.

Based on the TEs content in plant tissue, significant variations in the Cr and Ni content between celery and parsley are observed, while that was not the case for As and Cd (Figure 1). However, it can be noticed that As, Cd, and Ni accumulation was greater in celery, with the exception of Cr. Nevertheless, the majority of samples exceed the permissible limit for Cd and Cr, while the average of the As content was below the maximum allowed values in parsley, but not in celery (Figure 1). When it comes to the Cd content in celery, all analyzed samples exceed the limit of 0.2 mg/kg, while the average reached 0.72 mg/kg. A similar pattern was occurred in parsley, with a slightly lower average of 0.52 mg/kg. The differences in plant capacity to uptake and accumulated Cd was already reported in our previous study [16]. We concluded significantly higher Cd content in celery vs. parsley, especially in Cd-polluted soil, even with strong hyperaccumulation potential. Likewise, De Temmerman et al. [18] defined celery as a biomonitor for atmospheric pollution of certain TEs, since they observed that Cd and As content in its storage organ can reflect atmospheric deposition, while for Pb this is not observable. However, the main route of hazardous elements entrance to plants is the root system, therefore, we can suppose that plant contamination was most likely caused by polluted soil. As was previously observed, in the territory of Vojvodina Province, Serbia, hazardous elements (Cr, Ni, Cd, and As) loadings have been established in both soil and groundwater [19,20,21], consequently leading to a notable enrichment of TEs in various plant species and vegetables. In that sense, the consumption of vegetables grown on chemically loaded soil carries a considerable dietary risk and health implications. A Comparable situation occurred in Cr content, where the majority of samples exceeded limit values, but contrary to the other elements, a higher Cr concentration was detected in parsley. The total As content in parsley roots ranged from 0.06 to 0.42 mg/kg, while the highest registered concentration of As was almost four-fold greater than the limit (0.78 mg/kg) and was registered in celery. The literature data indicate that As absorption and its retention in roots can be a result of high phytochelatins accumulation and activation of detoxifying mechanisms, hence leading to its slow transport to the above-ground plant [22]. Along with the total As content, the proportion of inorganic arsenic (iAs) is an even more important trait, taking into account its harmful effect on human health. In this study, we were focused only on total elements content, but according to the literature data, it could be an assumed contribution of iAs in total As content. According to Monboonpitak et al. [23], the total As content in celery was 41.0 ng/g per dry weight of which 62.1% was inorganic As (iAs). Similarly, Fontcuberta et al. [23] recorded 62% of iAs in parsley in a total As content of 0.13 mg/kg. Contrary to other elements, both species showed Ni levels in an acceptable range for the study area, below the level of contamination, indicating that there was no danger of Ni-entry into the food chain in toxic amounts. Srinavas et al. [24,25] reported that Ni accumulation in vegetables and fruits grown in rural areas (uncontaminated) was in the range of 1.1–1.53 mg/kg, which is similar to our results.

### 2.2. Health Risk Assessment

Taking into account that total metal concentration in edible plants cannot provide sufficient data of an element’s impact on human health, various indices have been developed aiming to afford a better understanding of toxic substances’ influence on consumers. Therefore, we calculated the estimated daily intake (EDI) for each element and compared it to the maximum tolerable daily intake (MTDI) (Table 1). Data showed that the EDI values were below the MTDI for all analyzed elements.

**Figure 2:**
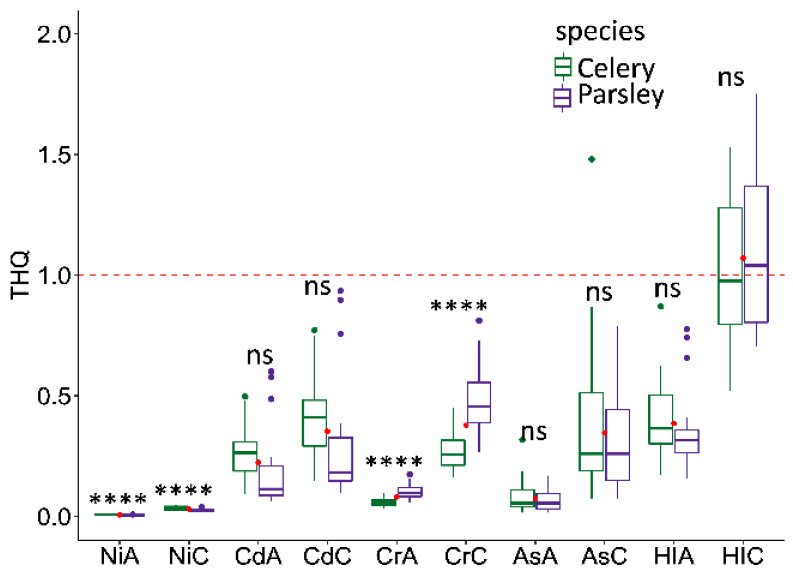
Target hazard quotients—THQ of Ni, Cd, Cr, As, and hazard index (HI) of celery and parsley. Values are calculated for both adults (A) and children (C) (*n* = 21). The line within the boxplots represent the median, the ends of the box show the upper (Q3) and lower (Q1) quartiles, the extreme lines show Q3 + 1.5 × IQR to Q1 − 1.5 × IQR, the dots beyond the extreme lines represent outliers, while red dots are the mean values of both groups. Red dashed line defines the limit of 1, where the values greater than 1 indicate potential non-cancerogenic risk. Significance codes: ns—non-significant; **** *p* ≤ 0.0001.

Following that, the target hazard quotients (THQ) were calculated for both adults and children, aiming to observe potential non-cancerogenic risks on citizens through the consumption of analyzed plant species. THQ, for each element, was below the limit of 1, indicating that there is no need for concern for inhabitants, in terms of the potential non-cancerogenic risk from consumption of selected vegetables (Figure 2). Comparable to the total element content, significant variation between the examined species was observed in THQ for Ni and Cr, but not for Cd and As, which was expected. Additionally, the hazard index (HI) was estimated as a sum of multiple hazardous substances, in order to give information on the possible synergistic effects of different elements’ influence on the resident’s health. In the adult population, HI was below 1 in all samples regarding their origin (Figure 2), so it can be concluded that the consumption of selected vegetables would not provoke any adverse health issues to the local population. However, the problem arises when it comes to children since the HI reached the limit of 1 in both analyzed species since around 40% of the samples surpassed the admissible level (Figure 2). In the adult population, Cd showed the highest contribution to the overall pollution index (see Supplementary Materials Table S1), contributing 51.2% in parsley and 59.1% in celery of the total hazard index. In respect to the children population, Cd was the leading pollutant in celery, contributing 37.5% to HI, while in parsley chromium showed the highest share in the overall pollution (43.6%). The literature data have demonstrated that significant health risks can be associated with Cd content in various vegetables, including celery and parsley [14,15,16,26]. However, according to the European Food Safety Authority (EFSA) consumption of leafy vegetables can contribute 3.9% of Cd exposure in the human diet, which represents a small quantity compared to grains (26.9%) and starchy vegetables and tubers (13.2%) [27]. Therefore, serious precautions should be taken, especially when it comes to children and vegetarians since crop intake represents only one of the multiple pathways to heavy metals exposure. The same statement was observed by Škrbić et al. [21], who analyzed the pollution status and health risks caused by heavy metals in the flooded soil and vegetables from typical agricultural regions in Vojvodina Province, Serbia. The same authors reported higher HI for children compared to adults (1.6 and 1.16, respectively) indicating that children are the more vulnerable category to TEs exposure.

### 2.3. Antioxidative Potential and Phenolic Status—SMA Model Fitting

**Figure 3:**
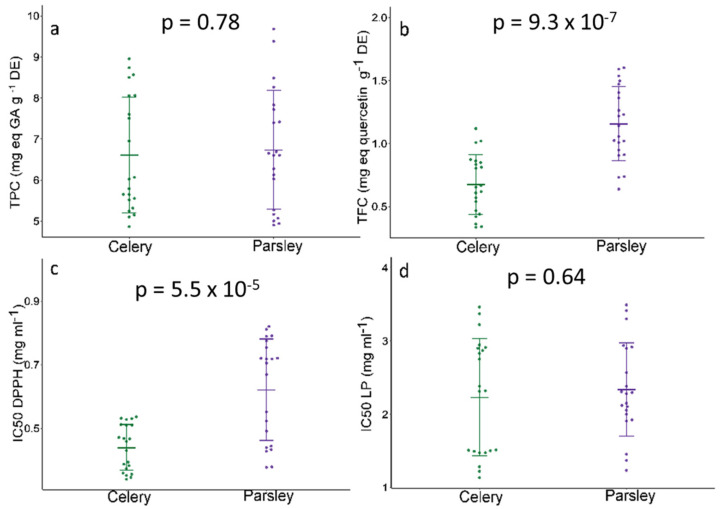
(**a**) TPC—total phenolic content; (**b**) TFC—total flavonoid content in celery and parsley root; (**c**) IC50 DPPH—DPPH-radical scavenging capacity; (**d**) IC50 LP—inhibition of lipid peroxidation of celery and parsley extract. Data are presented as the mean ± standard deviation. An unpaired *t*-test was used for comparing the mean between species (*n* = 21) and the *p*-value is assigned to each plot.

In spite of the TEs present, potential health-promoting roles of selected species were observed based on total phenolic (TPC) and flavonoid (TFC) contents, as well as on the basis of antioxidant activity (Figure 3). Both species showed a similar range of TPC (Figure 3a) in parsley root—the TPC ranged from 5.03 to 9.18 mg eq GA/g DE, while in celery roots, 5.04 to 8.50 mg eq GA/g DE, thus, no significant variation occurred between the examined species. Contrarily, TFC varied significantly between the species, with greater values observed in celery roots (Figure 3b).

Further, the detailed composition of phenols is presented by phenolic acids, flavonoids, and coumarins (Table 2). Among phenolic acids, the predominant compound was ferulic acid, which was followed by chlorogenic acids, where a slightly higher average was observed in parsley. The results of Babula et al. [28] also revealed the general arise of ferulic acid in the medicinal plant *Hypericum perforatum* exposed to elevated Cd concentrations. In addition, parsley was rich in protocatechuic, 2,5-dihydroxybenzoic, and p-coumaric acid, possessing significantly higher levels in comparison to those observed in celery. Among the flavonoids, apigenin and its glucosides dominated in the roots of both species. Apiin and apigenin-7-O-glucoside were significantly greater in the parsley root, with the exception of chrysoeriol which was more dominant in celery (Table 2). Apigenin is known as a naturally present flavonoid in various fruits and vegetables with an important role in suppressing cancer occurrences [29], which makes its presence in the human diet extremely desirable. According to Sung et al. [30], parsley can be labeled as one of the major sources of apigenin, withal apigenin is also an abundant flavone in chamomile flower, celery, and spinach. With respect to coumarins, the key compounds in our samples were scopoletin, umbelliferone, and aesculetin, which were especially dominant in celery roots (Table 2). The mentioned phytochemicals were previously identified as immuno-stimulating compounds with a strong contribution to immunomodulation, confirming their high therapeutic potential [2]. Along with the aforementioned, extensive studies have endorsed the information that a phenol-rich diet decreases oxidative stress, endorsing the valuable effects of flavonoids against cardiovascular diseases [1,30,31]. It was shown that besides their ability to scavenge free radicals in vivo, celery and parsley can even stimulate the antioxidant activity of processed foods [3]. Additionally, various secondary metabolites, such as polyphenols, are biosynthesized and accumulated in plant tissue as part of a defensive mechanism toward oxidative damage caused by ROS production, as a consequence of abiotic and biotic stresses [8]. Therefore, plants exhibiting phenolic production show better adaptability and overall fitness under changing environments [12].

**Figure 4:**
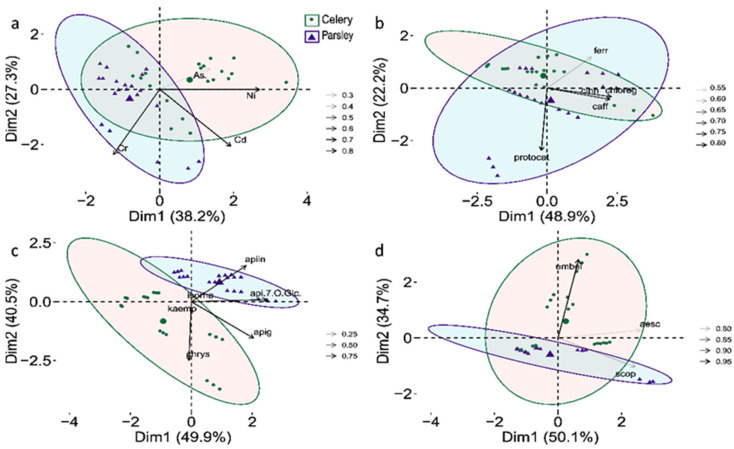
Multivariate analysis of trace elements content (TEs) and selected metabolites of celery and parsley; (**a**)—biplot of TEs across the first two examined dimensions (PC); (**b**)—biplot of phenolic acids, (**c**)—biplot of flavonoids; (**d**)—biplot of coumarins. Ellipses on biplot assume a multivariate normal distribution. Cos2 value is the square loadings for variables, presenting the quality of the representation of variables. Dim1 and Dim2 correspond to the first and second principal components. Variable symbols legend: As—arsenic concentration; Cd—cadmium concentration; Cr—chromium concentration; Ni—nickel concentration; aesc—aesculetin; apiina—apiin; apig—apigenin; api-7-O-Glc—apigenin-7-O-glucoside; caff—caffeic acid; cinn—cinnamic acid; chlorog—chlorogenic acid; chrys—chrysoeriol; isorha—isorhamnetin; ferr—ferulic acid; kaemp—kaempferol; protocat—protocatehuic acid; scop—scopoletin; umbel—umbelliferone.

Additionally, the antioxidant capacity of celery and parsley root extracts was evaluated by measuring the DPPH-radical scavenging capacity (DPPH) and inhibition of lipid peroxidation (LP), which are expressed as IC50 values (mg/mL) (Figure 3). The obtained results indicated powerful antioxidant activity in both celery and parsley roots, with significantly greater DPPH-radical scavenging capacity in celery, while similar LP inhibition was evident in both plant extracts. Despite the great variability in the quantitative composition of the identified phenolics between samples, in general, parsley showed a higher content of phenols and flavonoids. According to that, we have expected more efficient DPPH radical scavenging capacity in parsley because polyphenols act as reducing agents according to their redox properties, and can strongly contribute to free radical scavenging and antioxidant activity. However, when we expand our view to heavy metals content, it can be seen that celery accumulated higher doses of As, Cd, and Ni, therefore, a more potent free radical scavenging capacity in celery can be a result of high ROS production and oxidative stress occurred as a consequence of pollution. The influence of various heavy metals on the activation of defense machinery in terms of scavenging excess ROS production has been reported by a number of studies [32,33,34]. Apart from that, the slightly higher ability for lipid peroxidation inhibition observed in the celery extract can also be explained by higher TEs accumulation. To further explore this phenomenon, with an aim to estimate how TEs content scales against different compounds of secondary metabolism, we first run a principal component analysis (PCA) over TEs, followed by a PCA of phenolics, flavonoids, and coumarins data (Figure 4, Tables S2–S5).

**Figure 5:**
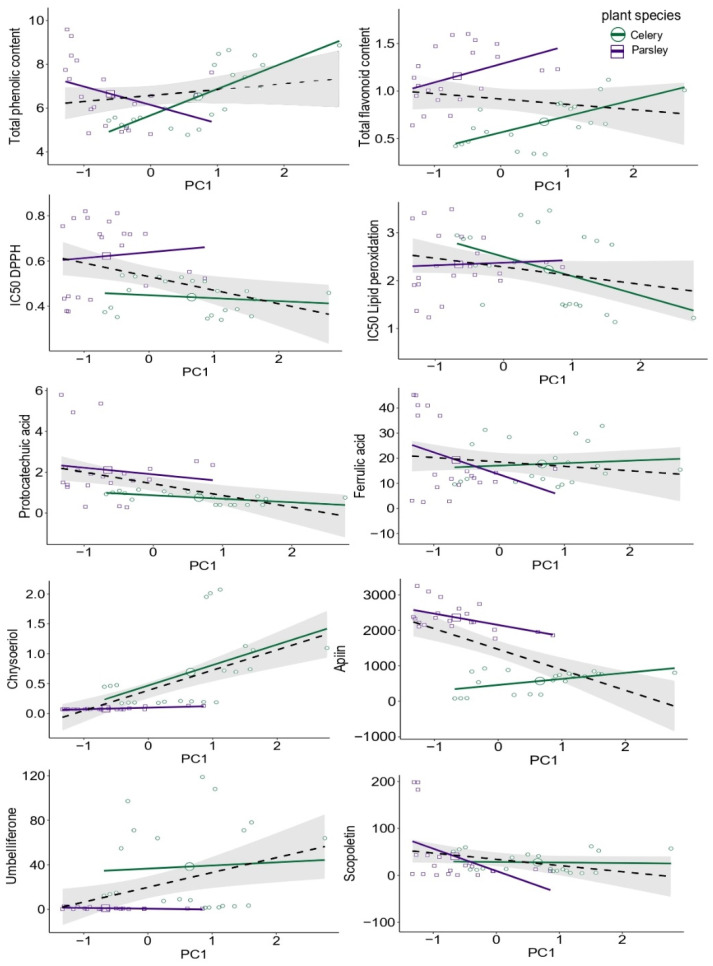
Standardized major axis (SMA) regression model of total phenolic, flavonoid content, IC50 of DPPH, and lipid peroxidation, as well as selected metabolites in relation to the variance contribution in PC1 from the trace elements data set. The black line reflects the overall SMA regression fit based on both species, celery, and parsley, while the gray area is showing the 95% confidence interval.

The given results are used for Standardized Major Axis (SMA) regression, which is an often utilized model since it can provide true information between two factors, assuming variation in both variables [17]. In the TEs data set, the majority of variance was weighted in the first two components explaining 65.5% in the overall variability, where PC1 contributed 38.2%, separating Cr from the rest of the TEs, which dominated in parsley (Figure 4). Therefore, for SMA, we used PC1 as a functional gradient, since it was a major axis explaining TEs variability and was sufficient to describe the essence of the data. Based on the PCA biplots of phenolics, flavonoids, and coumarins, we observed that the first two principal components explained 71.1% of the total variance in phenol acids, 90.4% in flavonoids, and 84.8% in the coumarins data set (Figure 4). Depending on the loadings of variables clustering across the biplot, we selected two compounds per group (phenolic acids, flavonoids, and coumarins) as factors used for SMA (Figure 5).

Within the phenolic acids data set, ferulic and protocatechuic acids were chosen as traits for SMA since they have the highest influence in the loadings separation, while they were negatively correlated to each other (Figure 4). Ferulic acid was positively correlated to both dimensions, while protocatechuic acid was strongly negatively correlated to the second dimension (see Supplementary Materials Table S4). The same selection pattern was used in the flavonoids PCA biplot; so, we selected chrysoeriol and apiin for SMA. In the coumarins data set, aesculetin and scopoletin showed strong positive correlations, so for further analysis, we used scopoletin, since it has higher contributions in PC2 and umbelliferone (see Supplementary Materials Table S5). Based on the SMA results, we have observed that the overall fit for total phenolic contents to PC1 showed an increasing trend with the elevation of TEs (β = 1.400225), where both species share a common slope (Table S6). However, the species showed significant differences in elevation and shift along the slope, where in celery, TPC was positively related to PC1, but in parsley, it occurred as an opposite trend (Figure 5). The SMA of the total flavonoid content revealed significant variation among groups, based on the slope (0.2626665 for parsley vs. 0.4761960 for celery), shift along the slope, as well as on elevation (Figure 5, Table S6). Further, a significant overall fit occurred in DPPH scavenging activity against TEs plotted on PC1, with significant variation along the slope (*p* = 0.00048134), and shift along the slope between groups, with no variation in elevation. However, LP showed no significant overall fit to PC1 (*p* = 0.09), with a similar common slope of (−0.94), while the species significantly differ in elevation, and shift along the slope (Figure 5). In general, model fitting was stronger in celery samples where a positive correlation between total phenolic, flavonoids content, and inhibition of lipid peroxidation in relation to the metal loadings in PC1, (*p* = 5.0881 × 10^−5^, 0.0012866, 0.036296, respectively), was recorded, suggesting the existence of an assumed relationship. When it comes to selected metabolites, the variation between species occurred in the slope, elevation (with the exception for protocatechuic acid *p* = 0.27), and slope shift across PC1 (see Supplementary Materials Table S7). All these confirm that the assumption of TEs present in plant tissue can cause fluctuations in the production and accumulation of the bioactive compounds, therefore, it can change the metabolic profile to some extent. It is reported that coumarins dominated in celery (Table 2), additionally, based on the SMA model a positive trend regarding PC1 in celery (β = 44.71397) can be noticed, while in parsley, it occurred as a weak negative trend based on the slope (β = −1.5026377). That clearly concludes the differences between the examined species, especially when it comes to the bioproduction of specific metabolites.

## 3. Materials and Methods

### 3.1. Vegetable Sampling

**Figure 6:**
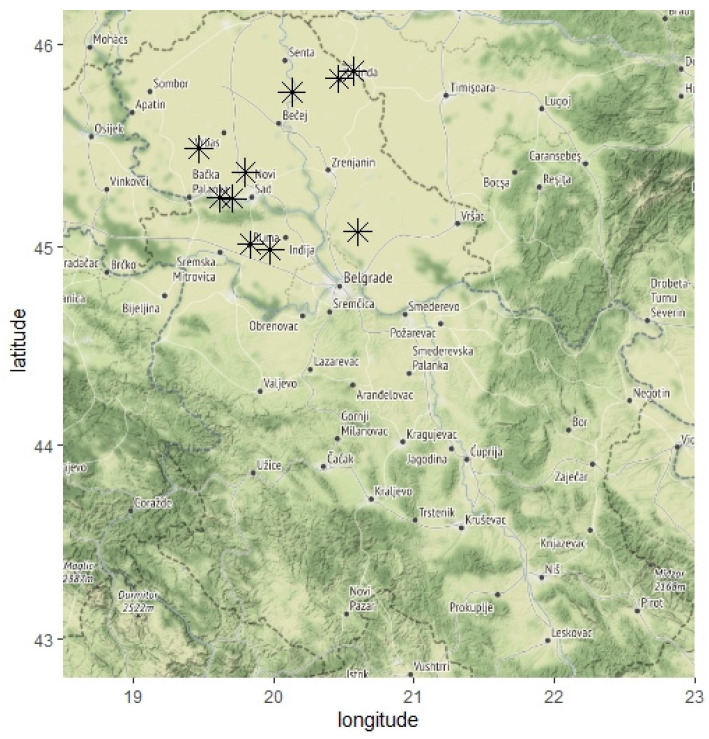
Location map of Vojvodina Province, Serbia. The sampling sites are indicated with asterisks.

Based on the known fact that the accumulation of bioactive compounds in plants is not consistent and can vary depending on ontogeny, physiological states, and various intrinsic and extrinsic factors, commercially available plants of parsley and celery on the green markets were analyzed. The roots of parsley (*Petroselinum crispum* var. *tuberosum* (Mill.) Fuss) and celery (*Apium graveolens* var. *rapaceum* L.) were obtained from individual producers, who cultivated them on agricultural land in the southern part of the Pannonian plain, Vojvodina Province, the northern part of Serbia, 45.2609° N, 19.8319° E (Figure 6). This part of Serbia is covered by agricultural land, and the soil type is chernozem; the climate is temperate continental; the average annual precipitation is around 670 mm and the average annual temperature is 11 °C, which are all favorable conditions for crops and vegetable production [19]. Based on our previous work [35], we selected plants grown at seven different localities that we previously defined as potentially polluted, and where common agricultural practices were applied, which implies the usage of fertilizers and pesticides, and frequent irrigation. The plants were collected in their full maturity in early October 2016 and were healthy based on their organoleptic traits.

### 3.2. Trace Elements Analyses

Prior to the analyses, fresh roots of celery and parsley were washed with distilled water aiming to remove any adsorbed pollution at the surface of the plants. Further, roots were chopped and oven-dried at 70 °C until constant weight, and ground and prepared for chemical analysis. Plant mineralization was carried out by digestion in 65% HNO_3_, following by the addition of 30% H_2_O_2_. After the complete mineralization process, the clear and colorless solution was filtered out through a Whatman filter paper into a 50 mL volumetric flask. Further, trace elements (As, Cd, Cr, and Ni) concentration in samples solutions were measured by an Inductively Coupled Plasma Mass Spectrometry (ICP/MS, Agilent Technologies 7700, Santa Clara, CA, USA.) according to the EPA method 6020B (SW-846). Standard stock solutions of these elements were of analytical reagent grade and were purchased from Merck Co. The instrument conditions were as follows: *m*/*z* analyzed: 75; RF power: 1600 W; sampling depth: 10 mm; operated in He mode: collision gas flow 5 mL He/min; concentric nebulizer used. The limit of detection (LOD) was ≥0.02 µg/L and was based on 3 times the standard deviation of 10 replicates, while the limits of quantification (LOQ) validated in our samples was 0.02 µg/L. The percentage of relative standard deviation (RSD) was below 10%. The trace elements concentration is presented as mg per kg of dry plant mass (mg/kg d.m.).

### 3.3. Human Health Risk Assessment

The health risk was expressed as the ingestion pathways of exposure, and was calculated based on the estimated daily intake (EDI), target hazard quotients (THQ), and hazard index (HI) according to the following equation:

EDI = (Ef × Ed × IR × Cm × Cf)/(BW × AT) × 10^−3^ (1)

where EDI—estimated daily intake (mg/day/kg BW) was calculated according to Gebeyehu and Bayissa [36] and compared to the Maximum Tolerable Daily Intake (MTDI); EF—exposure frequency (365 days/year); ED—exposure duration (65 years adults, 6 years children); IR—ingestion rate (for celery and parsley IR the value is set to 0.100 kg/day according to Harmanescu et al. [37]); Cm—concentration of metal in dry weight (mg/kg dw); Cf—conversion factor for fresh vegetable weight to dry weight (which is 0.085); BW—body weight (70 kg adults, 20 kg children); AT—average time exposure (23,725 days adults, 2190 children)

THQ = EDI/RfD(2)

where THQ—target hazard quotient indicates non-cancerogenic risk from consumption of potentially contaminated celery and parsley; EDI—is estimated daily intake (µg/g/day); RfD—is the oral reference dose (mg/kg/day). The RfD for Ni, Cd, Cr, and As are as follows: Ni–0.02, Cd–0.001, Cr–0.003, As–0.0003 [36].

(3) $$\mathrm{HI}\;=\;\sum_{n=1}^{i}\mathrm{THQn};\;i=1,\;2,\;3\ldots ,\;n$$

where HI—hazard index was calculated as the sum of target hazard quotients of each element. The THQ and HI values greater than 1 indicate potential non-cancerogenic effects from consumption of selected plant species to the local human population [38].

### 3.4. Extract Preparation

A sample of 30 g of dried roots was macerated 3 times with 40 mL of 80% methanol, with 6 h stirring, using fresh solvent each time. The macerate was separated from the rest of the plant material by filtering through filter paper. The extracts obtained were evaporated on a vacuum evaporator at a temperature below 45 °C until dry. The dry residue was then dissolved in DMSO so that the final concentration was 300 mg/mL.

### 3.5. Total Phenolic and Total Flavonoid Content

The total phenolic content was measured by the method of Singleton et al. [39], adapted for 96-well microplates. The results were expressed as milligrams of gallic acid equivalents per gram of dry extract (mg eq GA/g DE). The total flavonoid content was determined by the aluminum chloride colorimetric method [40]. All samples were made in triplicate, and the mean values of flavonoid content were expressed as milligrams of quercetin equivalents per gram of dry extract (mg eq quercetin/g DE).

### 3.6. Quantitative LC-MS/MS Analysis of the Selected Phenolics

The content of 44 selected phenolic compounds (14 phenolic acids, 25 flavonoids, 3 coumarins, and 2 lignans) was investigated by LC-MS/MS [41]. Standards of the compounds were purchased from Sigma-Aldrich Chem (Steinheim, Germany), Fluka Chemie GmbH (Buchs, Switzerland), or from ChromaD.e. (Santa Ana, CA, USA). An Agilent 1200 series liquid chromatograph, coupled with an Agilent series 6410B electrospray ionization triple-quadrupole mass spectrometer and controlled by MassHunter version B.03.01 software was used for the analysis. Analytes were separated using a Zorbax Eclipse XDB-C18 4.6 mm × 50 mm × 1.8 μm (Agilent Technologies) reversed-phase column.

### 3.7. Antioxidant Activity

The antioxidant potential was determined using the DPPH scavenging activity and lipid peroxidation (LP) inhibition assay. Plant extracts were tested for DPPH radical scavenging activity according to Soler-Rivas et al. [42]. The ability of extracts to inhibit lipid peroxidation was evaluated by the spectrophotometric TBA assay [43]. Linseed oil obtained from linseed by hexane extraction was used as a source of polyunsaturated fatty acids (69.7% linolenic, 13.5% linoleic acid, as determined by GC-MS). All samples were made in triplicate and the IC50 values were calculated.

### 3.8. Data Analyses

All analyses were performed within the R Programing Environment [44]. The majority of data were expressed as the mean ± standard deviation (SD) of three replicates (*n* = 3 per each locality, 21 in total), while an unpaired *t*-test was performed to identify variability between species. An exception was made for phenols, where data were expressed as the median ± the mean absolute standard deviation (MAD) of three replicates (*n* = 21), and differences between species were evaluated by one-way ANOVA. Dot plots and boxplots were prepared by using the ggplots2 package, while the map was created using the maps package. Following the univariate analyses, we applied principal component analysis (PCA) over TEs, phenolics, flavonoids, and coumarins, aiming to observe a clearer and more complete picture through integrated evaluation of the analyzed variables. Multivariate PCA was conducted on a correlation matrix with previously scaled and centered values in the dataset, using the following packages: ade4, vegan, and factoextra, and then we applied the standardized major axis (SMA) model using the smatr package. In the SMA analysis, β estimates the line describing the bivariate scatter of Y and X. We used SMA regression aiming to understand how the TEs presence affects the metabolic response; thus, we tested hypotheses about the nature of this relationship and how it varies between species.

## 4. Conclusions

Based on all obtained results, we can conclude that there is a direct link between TEs present in plant tissue and the production of a wide range of secondary metabolites. Relatively higher As, Cd, and Ni content were recorded in celery, which was followed by greater DPPH radical scavenging activity and elevation of coumarins content correlated to the TEs presence. On the contrary, parsley accumulated chromium to a greater extent, as well as flavonoids, such as apigenin and its glucosides. The SMA regression model showed species-specific responses in bioactive compounds production corresponding to metals loadings in plant tissue. Celery showed a significant positive correlation between total phenolic and flavonoids content, and inhibition of lipid peroxidation in relation to metal loadings, while that was not the case in parsley. The results of this study imply the need for detailed analyses of metabolic profiles regarding the potential pollution, aiming to define plant quality more in-depth. Such data will fulfill the current knowledge and can improve agricultural production, food quality, and economic gain. Furthermore, special attention should be given prior to recommending the usage of certain plants in the human diet. With respect to that, future studies should be focused on defying threshold values of certain heavy metals, especially in edible parts of vegetables including medicinal plants and crops that can up- or down-regulate bioactive compounds production. Furthermore, future perspectives should be turned towards the production of genotypes with a lower potential for toxic elements accumulation, therefore, the health benefits will be more prominent.

## Figures and Tables

**Table 1:** Estimated daily intake—EDI (mg/kg/bw) of Ni, Cd, Cr, and As in celery and parsley. Values are presented as the mean (*n* = 21) ± standard deviation (SD) and compared with the tolerable daily intake (MTDI) mg/kg.

Celery					Parsley				MTDI
Adults			Children		Adults		Children		
Mean		SD	Mean	SD	Mean	SD	Mean	SD	
Ni	1.53 × 10^−4^	3.18 × 10^−5^	8.73 × 10^−4^	7.14 × 10^−4^	1.12 × 10^−4^	5.24 × 10^−4^	5.24 × 10^−4^	1.14 × 10^−4^	0.1–0.3
Cd	2.64 × 10^−4^	1.10 × 10^−4^	4.65 × 10^−4^	4.11 × 10^−4^	1.83 × 10^−4^	1.60 × 10^−4^	2.95 × 10^−4^	2.46 × 10^−4^	0.02–0.07
Cr	1.79 × 10^−4^	5.13 × 10^−5^	6.91 × 10^−4^	8.36 × 10^−4^	3.06 × 10^−4^	8.65 × 10^−5^	1.37 × 10^−3^	1.43 × 10^−3^	0.035–0.2
As	2.43 × 10^−5^	2.07 × 10^−5^	1.54 × 10^−4^	1.53 × 10^−4^	2.02 × 10^−5^	1.31 × 10^−5^	2.15 × 10^−4^	1.12 × 10^−4^	0.13

**Table 2:** Phenolic compounds content (µg/g dry extract) in celery and parsley root. Data are presented as the median ± the mean absolute standard deviation (*n* = 21). One-way ANOVA was used for comparison of the median between species.

	Celery		Parsley		ANOVA	
	Median	MAD	Median	MAD	*p* Values	Sign ^1^
p-OH-benzoic acid	2.3	0	LOD	0	0.08	.
cinnamic acid	2.4	0.37	2.18	0.75	0.13	ns
protocatechuic acid	0.77	0.35	1.79	0.64	2.00 × 10^−4^	***
2,5-dihydroxybenzoic acid	LOD	0	2.68	0.56	4.30 × 10^−5^	***
p-coumaric acid	LOD	0	1.55	0.66	1.10 × 10^−5^	***
caffeic acid	2.46	0.55	2.84	0.76	0.08	.
ferulic acid	13.5	7.22	15.4	6.09	0.65	ns
chlorogenic acid	9.14	6.4	9.83	12.9	0.29	ns
apigenin	0.89	0.96	1.83	0.96	0.51	ns
apigenin-7-O-glucoside	0.8	0.69	2.32	0.87	3.80 × 10^−5^	***
chrysoeriol	0.46	0.41	0.07	0	7.89 × 10^−5^	***
apiin	705.41	207.23	2266.3	303.65	<2.2× 10^−16^	***
umbelliferone	13.5	17.8	0.56	0.53	1.25 × 10^−4^	***
aesculetin	0.37	0.4	0.1	0	0.31	*
scopoletin	13.9	13	13.0	15.3	0.39	ns

Data are presented as the median ± the mean absolute standard deviation (MAD), (*n* = 21). One-way ANOVA was used for comparison of the means between species.^1^ Signif. codes: 0 ‘***’ 0.001 ‘*’ 0.05 ‘.’ 0.1 ‘ns’ 1. LOD—values lower than limit of detection. The following metabolites were lower than the limit of detection in all analyzed samples: o-coumaric acid, vanillic acid, gallic acid, syringic acid, 3,4-dimethoxycinnamic acid, synapic acid, daidzein, genistein, kaempferol, luteolin 7-O-glucoside, catechin, epicatechin, quercetin, isorhamnetin, myricetin, matairesinol, secoisolariciresinol, vitexin, baicalin, quercitrin, kaempferol-3-O-glucoside, epigallocatechin gallate, hiperoside, amentoflavone, rutin, quercetin-3-O-glucoside, baicalein, luteolin, naringenin.

## Data Availability

All data are available upon request. The RStudio packages used in study are indicated in the manuscript, while all codes are available upon request.

## Supplementary Materials

The following Tables S1–S7 are available online at https://www.mdpi.com/article/10.3390/plants10091785/s1, Table S1: Trace elements (Cd, Ni, As and Cr) contribution to overall pollution index. Values are calculated for both adults (A) and children and presented as mean (*n* = 21). Table S2: Output from PCA on trace elements (TEs) dataset. Table S3: Output from PCA on flavonoids traits dataset. Table S4: Output from PCA on phenolics traits dataset. Table S5: Output from PCA on coumarins traits dataset. Tables S6 and S7: Statistical results of the standardized major axis (SMA) regression.
